# Impact of the calcium form of β-hydroxy-β-methylbutyrate upon human skeletal muscle protein metabolism

**DOI:** 10.1016/j.clnu.2017.09.024

**Published:** 2018-12

**Authors:** D.J. Wilkinson, T. Hossain, M.C. Limb, B.E. Phillips, J. Lund, J.P. Williams, M.S. Brook, J. Cegielski, A. Philp, S. Ashcroft, J.A. Rathmacher, N.J. Szewczyk, K. Smith, P.J. Atherton

**Affiliations:** a MRC-ARUK Centre for Musculoskeletal Ageing Research, National Institute for Health Research Nottingham Biomedical Research Centre, Clinical, Metabolic and Molecular Physiology, University of Nottingham, Royal Derby Hospital Centre, Derby, UK; b Metabolic Technologies, Inc, Iowa State University Research Park, 2711 S. Loop Drive, Ste 4400, Ames, IA 50010, USA; c School of Sport, Exercise and Rehabilitation Sciences, University of Birmingham, Birmingham, UK

**Keywords:** β-Hydroxy-β-methylbutyrate, Skeletal muscle, Protein metabolism, Anabolism, HMB, β-hydroxy-β-methylbutyrate, FA-HMB, free acid HMB, CaHMB, calcium HMB, MPS, muscle protein synthesis, MPB, muscle protein breakdown, A-V, arterio-venous, PA, postabsorptive, BCAA, branched chain amino acids, α-KIC, alpha-ketoisocaproate, GC–MS, gas chromatography–mass spectrometry, GC-C-IRMS, gas chromatography–combustion–isotope ratio mass spectrometry

## Abstract

**Background & aims:**

β-hydroxy-β-methylbutyrate (HMB) is purported as a key nutritional supplement for the preservation of muscle mass in health, disease and as an ergogenic aid in exercise. Of the two available forms of HMB (calcium (Ca-HMB) salt or free acid (FA-HMB)) – differences in plasma bioavailability have been reported. We previously reported that ∼3 g oral FA-HMB increased muscle protein synthesis (MPS) and reduced muscle protein breakdown (MPB). The objective of the present study was to quantify muscle protein metabolism responses to oral Ca-HMB.

**Methods:**

Eight healthy young males received a primed constant infusion of 1,2 ^13^C_2_ leucine and ^2^H_5_ phenylalanine to assess MPS (by tracer incorporation in myofibrils) and MPB (via arterio-venous (A-V) dilution) at baseline and following provision of ∼3 g of Ca-HMB; muscle anabolic (MPS) and catabolic (MPB) signalling was assessed via immunoblotting.

**Results:**

Ca-HMB led a significant and rapid (<60 min) peak in plasma HMB concentrations (483.6 ± 14.2 μM, *p* < 0.0001). This rise in plasma HMB was accompanied by increases in MPS (PA: 0.046 ± 0.004%/h, CaHMB: 0.072 ± 0.004%/h, *p* < 0001) and suppressions in MPB (PA: 7.6 ± 1.2 μmol Phe per leg min^−1^, Ca-HMB: 5.2 ± 0.8 μmol Phe per leg min^−1^, *p* < 0.01). Increases in the phosphorylation of mTORc1 substrates i.e. p70S6K1 and RPS6 were also observed, with no changes detected in the MPB targets measured.

**Conclusions:**

These findings support the pro-anabolic properties of HMB via mTORc1, and show that despite proposed differences in bioavailability, Ca-HMB provides a comparable stimulation to MPS and suppression of MPB, to FA-HMB, further supporting its use as a pharmaconutrient in the modulation of muscle mass.

## 1 Introduction

The maintenance of muscle mass is vital for maintaining health and well-being across the life course. Indeed the loss of muscle mass due to “healthy” ageing, or disease (i.e. cancer, COPD, diabetes, kidney or liver disease), is an influential factor in functional impairment, loss of independence, the onset of cardiovascular/metabolic disease and an increased mortality risk [1], [2], [3]. Muscle mass is regulated by the diurnal balance between muscle protein synthesis (MPS) and muscle protein breakdown (MPB) [4]. Loss of muscle protein in fasted periods (i.e. in between meals and overnight where MPB > MPS) is offset via intake of nutrition causing a stimulation of MPS [5] and an insulin mediated suppression of MPB [6]. It has been known for ∼25 y that essential amino acids (EAA) are the primary nutrients driving increases in MPS after feeding [4], [7], with the branched chain amino acid (BCAA) leucine acting as an anabolic ‘signal’ and being key to this effect [8], [9]. However, as a BCAA, leucine undergoes catabolism within muscle, metabolites of leucine could therefore contribute to the anabolic responses to leucine.

In the sarcoplasm and mitochondria, leucine is transaminated to α-ketoisocaproate (α-KIC). The majority of α-KIC is transported to the liver where it undergoes irreversible oxidation for the production of the energy substrates acetoacetate and acetyl Co-A [10]. However, in both muscle and liver, some α-KIC is metabolized via KIC-dioxygenase (more commonly called 4-hydroxyphenylpyruvate dioxygenase; HPD) to produce β-hydroxy-β-methylbutyrate (HMB) [10], [11], with a recent study in rats showing active conversion of ^14^C-leucine to HMB *in vivo*, with appearance of ^14^C-HMB in both plasma and urine following an oral dose of ^14^C Leucine [12]. Of all the leucine metabolites formed, the efficacy of HMB in producing anabolic/anti-catabolic effects is perhaps most compelling. HMB induces upregulation of mTOR, p70S6K1, 4EBP1 and an associated increase in protein synthesis in C2C12 murine myotubes [13], whilst also showing an ability to suppress ubiquitin-proteasomal regulated MPB [14], [15] and inhibition of myonuclear apoptosis by antagonizing mitochondrial-associated caspases [16]. In humans, HMB supplementation has been shown to reduce muscle wasting in clinical populations (Cancer [17], COPD [18], AIDS [19]), whilst also increasing lean body mass and protein turnover chronically in ageing cohorts when supplemented alongside the AAs arginine and lysine [20], in addition to attenuating muscle loss during a period of 10 days of bed rest [21], however the efficacy of HMB in these situations is confounded by the inclusion of other AAs within the supplementation. Moreover, its efficacy as a supplement for the preservation of muscle mass was recently highlighted in a meta-analyses [22], with a positive relationship also being identified between endogenous HMB concentrations and appendicular lean mass and grip strength [23].

Much of the research to date regarding HMB has been performed with the use of HMB in its calcium salt form (Ca-HMB), however recently HMB in its free acid form (FA-HMB) has been shown to provide improved bioavailability [24]. Indeed, pharmacokinetic studies have demonstrated that FA-HMB, independent of the form of administration (i.e. gel [24], capsule [25] or dissolved in water [25]), provided a greater plasma bioavailability compared with Ca-HMB, with a greater and more rapid rise to peak plasma HMB levels following administration [24], [25]. Moreover, we have recently shown that ∼3 g oral FA-HMB robustly stimulates MPS (and suppresses MPB independent of insulin) to a similar extent to 3 g of leucine in young men [8]. Distinct bioavailability of Ca-HMB vs. FA-HMB [25], [26] could lead to distinct effects upon muscle protein turnover, i.e. is there a relationship between plasma bioavailability and the effects of HMB, which is crucial knowledge for research in this area. The aim of this study was to independently investigate the effect of oral Ca-HMB upon muscle protein metabolism, i.e. stimulation of MPS and suppression of MPB, in relation to that empirically seen with the more bioavailable FA-HMB form.

## 2 Materials and methods

### 2.1 Ethical approval

All studies were conducted in accordance with the Declaration of Helsinki, with ethical approval obtained from the University of Nottingham Ethics Committee. Volunteers were recruited from the local Derbyshire area via postal advertisement. Following recruitment and before inclusion in the project all volunteers were additionally screened by a physician to exclude for any metabolic, respiratory, cardiovascular/vascular or other symptoms of ill health. All volunteers provided written informed consent before participation in the study.

### 2.2 Subject characteristics and study design

**Fig. 1:**
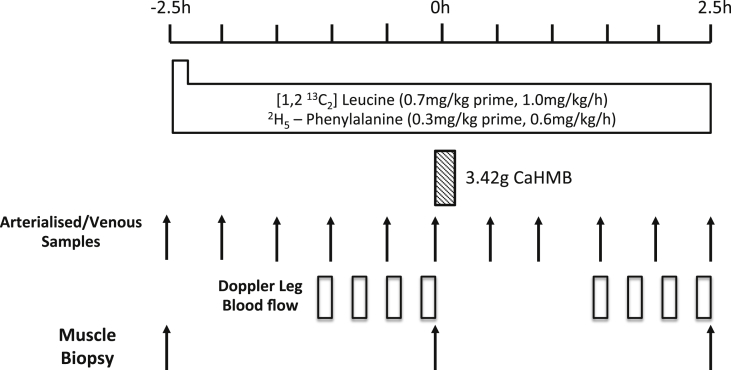
Study schematic for assessing the effects of Ca-HMB on human skeletal muscle metabolism.

Young healthy male volunteers (*n* = 8, age 26 ± 2 y, BMI 27.6 ± 1.2  kg m^−2^) were recruited to participate in the study. Volunteers were asked to refrain from heavy exercise for the 72 h before the study. On the morning of the study (∼08:30 h), following an overnight fast volunteers had an 18 g cannula inserted into the antecubital vein of one arm to enable tracer infusion, a retrograde 22 g cannula inserted to sample arterialized blood, and a femoral vein cannulae to enable sampling of arterio-venous blood for MPB (rate of appearance) measures. Following collection of the first muscle biopsy, a primed, continuous infusion of [1,2-^13^C_2_]Leu (0.7 mg kg^−1^ prime, 1 mg kg h^−1^ continuous infusion) tracer and L-[ring-^2^H_5_]-phenylalanine (0.3 mg kg^−1^ prime, 0.6 mg kg h^−1^ continuous infusion) (99 Atoms %, Cambridge Isotopes Limited, Cambridge, MA-US) was started and maintained until the end of the study (+2.5 h). During the first 2.5 h period we gathered postabsorptive/fasted measurements, the volunteers then consumed 3.42 g of Ca-HMB (equivalent to 2.74 g of FA-HMB) dissolved in ∼100 ml of water (Metabolic Technologies, Inc., Ames, IA, USA), such that we were able to gather measures of the effects of Ca-HMB on protein turnover over the subsequent 2.5 h (see Fig. 1). Muscle biopsies (∼150 mg) were taken from the mid *m.vastus lateralis*, under sterile conditions using a local anesthetic (1% lidocaine) and the conchotome technique [27]. Blood sampling, for the measure of MPB via A-V balance and the quantification of plasma HMB concentrations, was performed every 30 min.

### 2.3 Plasma phenylalanine and HMB analyses

Plasma phenylalanine was analysed as described in [28]. Plasma was deproteinised with 1 ml ice cold ethanol following treatment with urease. After incubation on ice for 20 min, samples were centrifuged and supernatants evaporated to dryness under nitrogen. Following lipid extraction via ethyl acetate, samples were derivatized as their t-BDMS esters before analysis via gas chromatography-mass spectrometry (GC–MS). Plasma HMB was extracted from plasma with ethyl ether, then back-extracted into 0.1 M phosphate buffer, dried and analysed by GC–MS as described by Nissen et al. [29]. There was insufficient blood volume for measurement of HMB at all time points.

### 2.4 Measurement of myofibrillar MPS

Muscle samples (∼20–30 mg) were homogenised in ice cold homogenisation buffer (50 mM Tris–HCl (pH 7.4), 50 mM NaF, 10 mM β-glycerophosphate disodium salt, 1 mM EDTA, 1 mM EGTA, 1 mM activated Na_3_VO_4_ (all Sigma–Aldrich, Poole, UK)), followed by centrifugation (10,000g) to pellet the non-soluble components; the pellet was washed with homogenisation buffer and solubilised in NaOH to facilitate the separation of the soluble myofibrillar fraction from the insoluble collagen fraction by subsequent centrifugation. The soluble myofibrillar fraction was then removed and precipitated using 1 M perchloric acid (PCA) and pelleted by centrifugation. Following washing of the myofibrillar pellet with 70% ethanol, the protein-bound AA were released by acid hydrolysis using 0.1 M HCl and 1 ml of Dowex ion-exchange resin (50W-X8-200) overnight at 110 °C. AA were further purified by ion-exchange chromatography on Dowex H^+^ resin columns and derivatized to their N-acetyl-N-propyl esters. The samples were analysed using gas chromatography combustion isotope-ratio mass spectrometry (GC-C-IRMS) on a Delta Plus XL (Thermo Fisher Scientific, Hemel Hempstead, UK) [30]. The fractional synthetic rate (FSR) of the myofibrillar proteins was calculated using a standard precursor-product method:

$$\text{FSR}\;\left(\%\;h^{-1}\right)=\delta E_{\text{m}}/E_{\text{p}}\times 1/t\times 100$$

where δ*E*_m_ = the change in the [1,2-^13^C_2_]Leu enrichment in atoms per excess (APE) between subsequent biopsies, separated by the time period (*t*), and *E*_p_ = the mean enrichment over the same time period (*t*) of the precursor for protein synthesis, (plasma α-KIC was used as a surrogate for leucyl-tRNA [31]), following derivatization to its t-butyldimethylsilyl (tBDMS)-quinoxalinol form.

### 2.5 Measurement of MPB

MPB was calculated as previously described by arteriovenous (A–V) dilution of the [^2^H_5_]-phenylalanine tracer [32]:

$$\text{MPB}\;\left(\text{μmol}\;\text{Phe}\;\text{per}\;\text{leg}\;{\text{min}}^{-1}\right)=\left[\left(E_{\text{a}}/E_{\text{v}}\right)-1\right]\times \text{Ca}\times \text{BF}$$

where *E*_a_ and *E*_v_ are the steady state [^2^H_5_] phenylalanine enrichment values of arterialized and venous samples, respectively, Ca is the mean [^2^H_5_] phenylalanine concentration in arterialized plasma, and BF is arterial blood flow in ml leg^−1^, adjusted for plasma (haematocrit).

### 2.6 Measurement of anabolic and catabolic signalling via immunoblotting

Muscle (∼25 mg) was powdered on dry ice using a Cellcrusher™ tissue pulverizer (Cellcrusher Ltd, Cork, Ireland) and prepared as described [33]. Equal amounts of protein (30 μg) were boiled for 5 min in 1 × Laemmli buffer and separated on 10–15% gels by SDS-PAGE for 1 h. Following electrophoresis, proteins were transferred to a BioTrace nitrocellulose membrane (Pall Life Sciences, Pensacola, FL, USA) at 100 V for 1 h. Membranes were incubated overnight with the following 1° antibodies: phospho-S6K1 Thr389 (#9206), total 70 kDa S6 protein kinase (p70S6K1; #2708), phospho-S6 ribosomal protein Ser240/244 (#5364), total S6 (#2217), phospho-protein kinase B (Akt) Ser473 (#4060), phospho-Akt Thr308 (#2965), total Akt (#4691) all from Cell Signalling Technology, Danvers, MA, USA. Total MuRF1 (sc-398608) was from Santa Cruz Biotechnology Inc, Heidelberg, Germany, and MAFbx (ab92281) was from AbCam, Cambridge, UK. Immobilon chemiluminescent HRP substrate (Merck Millipore, Watford, UK) was used to quantify protein content following IgG binding, visualized on a G:BOX Chemi XT4 imager using GeneSys capture software (Syngene UK, Cambridge, UK). Imaging and band quantification were carried out using a Chemi Genius Bioimaging Gel Doc System (Syngene). Phosphorylated proteins were normalised to the relevant total protein, whilst total protein content was normalised to ponceau staining.

### 2.7 Statistical analyses

Descriptive statistics confirmed normal data distribution using a Kolmogorov–Smirnov test. Data are shown as mean ± SEM with the analysis of primary endpoints (e.g. MPS, MPB and signalling) using paired *t*-tests; repeated measures ANOVA was used for plasma HMB analyses with Bonferroni correction (Graph Pad, Version 5, La Jolla, San Diego, CA). The alpha level of significance was set at *p* < 0.05.

## 3 Results

### 3.1 Plasma HMB bioavailability

**Fig. 2:**
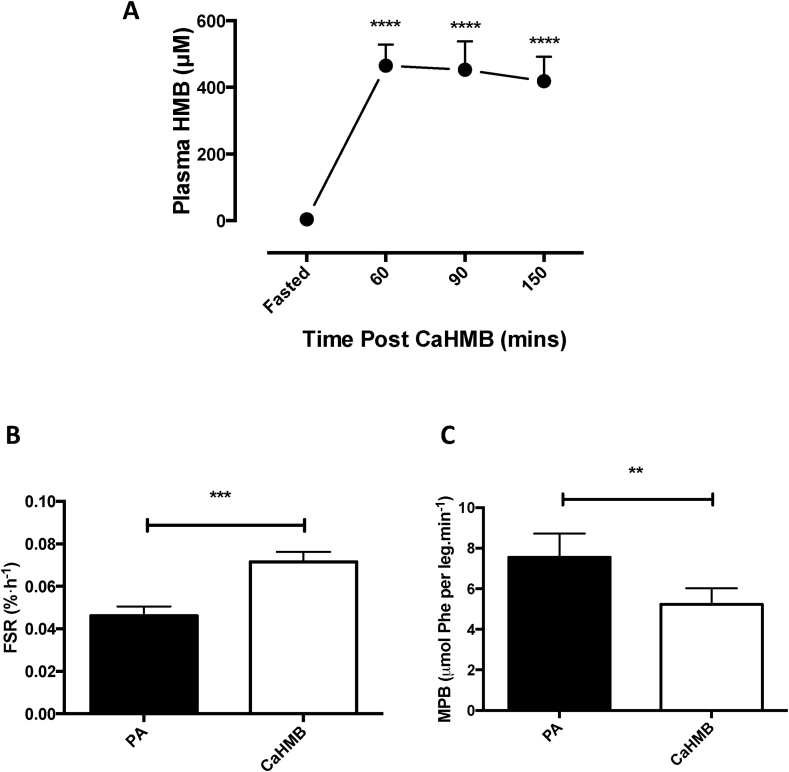
A) Bioavailability of HMB following oral provision of Ca-HMB measured by plasma HMB concentrations. ****Significantly different from fasted, *p* < 0.0001. Effects of Ca-HMB on B) human skeletal muscle myofibrillar protein synthesis (FSR) and C) muscle protein breakdown. Significant difference from post-absorptive (PA), ***p < 0.001, **p < 0.01.

Plasma HMB increased rapidly from fasted levels of 4.4 ± 1.3 μM peaking at 483.6 ± 14.2 μM after 60 min (*p* < 0.0001; Fig. 2). Plasma HMB remained markedly elevated over fasted levels up to 150 min (421.8 ± 29.6 μM, *p* < 0.0001), indicating robust bioavailability of HMB in this form and through this method of delivery.

### 3.2 Effects of Ca-HMB on muscle protein metabolism

Increases in plasma HMB bioavailability were accompanied by increases in myofibrillar FSR, which increased from post-absorptive levels over the experimental 2.5 h period, with an approximate doubling of MPS (PA: 0.046 ± 0.004%/h, Ca-HMB: 0.0715 ± 0.004%/h, *p* < 0.001; Fig. 2B). This increase was comparable to that previously reported with the FA-HMB [8]. Ca-HMB also elicited significant decreases in MPB (Fig. 2C), approximately halving the Ra of phenylalanine per leg (PA: 7.6 ± 1.2 μmol Phe per leg min^−1^, Ca-HMB: 5.2 ± 0.8 μmol Phe per leg min^−1^, *p* < 0.01), again comparable to FA-HMB [8].

### 3.3 Effects of Ca-HMB on anabolic and catabolic signalling

**Fig. 3:**
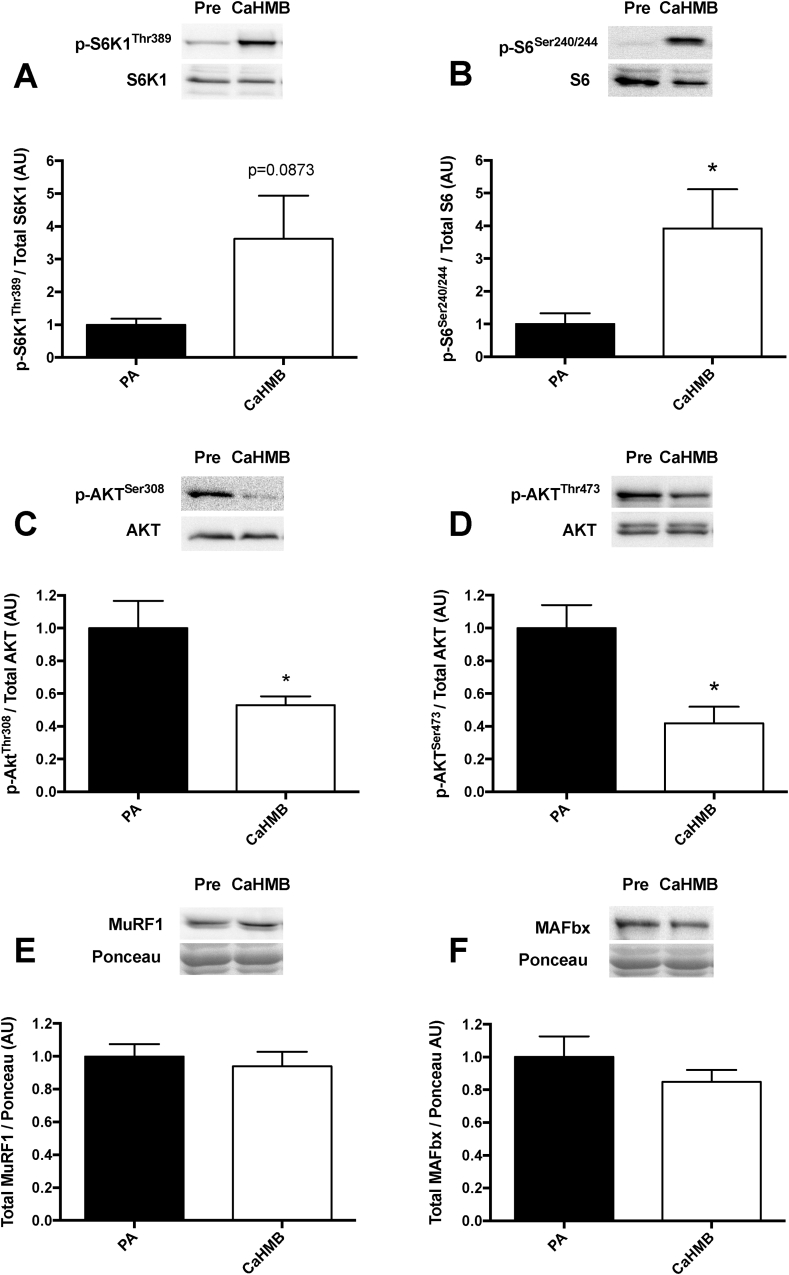
Signalling responses of A) p-p70S6K1^Thr389^, B) p-RPS6^Ser240/244^, C) p-Akt^Ser308^, D) p-Akt^Thr473^, E) Total MuRF1 and F) Total Mafbx to oral provision of CaHMB. Significant difference from post-absorptive (PA), *p < 0.05.

Provision of Ca-HMB led to a significant increase in the phosphorylation of the mTOR signalling targets p70S6K (∼3 fold, *p* = 0.0873 – 2 tailed, 0.0436 – 1 tailed; Fig. 3A) and RPS6 (∼3 fold, *p* < 0.05 – 2 tailed; Fig. 3B). This was accompanied by a significant decrease in the phosphorylation of Akt at serine 308 and threonine 473 (both ∼1 fold, *p* < 0.05 Fig. 3C and D). There was no effect on key muscle catabolism proteolytic markers (MuRF1/MAFbx; Fig. 3E and F).

## 4 Discussion

**Fig. 4:**
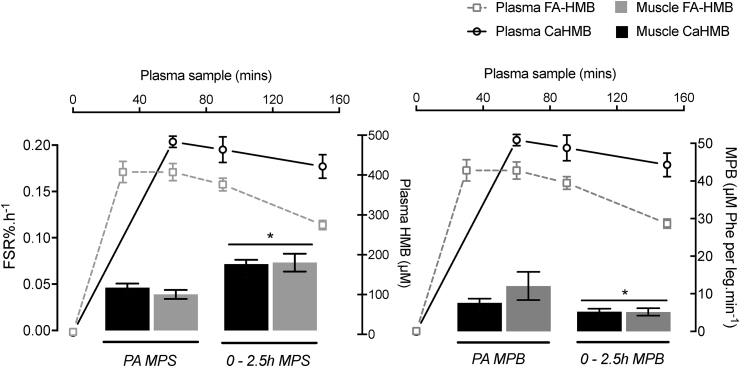
Summary comparison of anabolic and catabolic responses to oral provision of 3.42 g of Ca-HMB (equivalent to 2.74 g of FA-HMB) and 2.42 g FA-HMB (FA-HMB data taken from Wilkinson et al. [8]). Both forms of HMB result in an equivalent stimulation of MPS and suppression of MPB (* represents significant change from postabsorptive) highlighting potent (and possibly maximal) anabolic effects from a single acute oral dose of HMB independent of calcium or free-acid form.

The bioavailability of Ca-HMB vs. FA-HMB has been somewhat controversial in rats [26] vs. humans [25]. We found that Ca-HMB had a robust level of bioavailability, with plasma concentrations peaking after 60 min and remaining elevated throughout the study (see Fig. 2A). In the present study Ca-HMB was provided mixed in a small volume of water (∼100 ml). In a recent study by Fuller and colleagues to assess this bioavailability issue in humans further, it was found that there was a difference in bioavailability when Ca-HMB was provided dissolved in water compared to capsule form, with greater and more rapid appearance of Ca-HMB in plasma with the former [25]. However, when directly compared with FA-HMB, the plasma bioavailability of both Ca-HMB delivery methods was less than FA-HMB, with a significantly earlier peak in concentration at 30 min with FA-HMB [25], in direct contrast to that observed in rats [26]. Despite this proposed difference in bioavailability, it is clear that HMB even in its calcium form, evokes a robust (perhaps maximal) stimulation of MPS in healthy young males, and that this anabolic potential provides further support for its role as an important pharmaconutrient for the modulation of muscle mass in both health and disease. The optimal dose of Ca-HMB to achieve maximal ideal muscle mass and strength gains was determined to be 3 g/d (0.38 mg kg^−1^ d^−1^) and was delivered in 2–3 smaller daily doses [34], [35]. While no dose response studies have been conducted for MPS/MPB, we speculate that irrespective of bioavailability differences, provision of equated doses of either Ca-HMB or FA-HMB (at least in the ∼3 g range) is sufficiently over the threshold to saturate effects on MPS/MPB (Fig. 4). It is tempting to speculate that smaller doses of HMB are required to maximize anabolic effects; this premise might make sense given the small amount created upon leucine consumption, *in vivo* [10], [11] and smaller 1–1.5 g doses of Ca-HMB or FA-HMB have been used in practice to modulate muscle mass and strength in both health and disease [19], [34], [35], [36]. Clearly further studies are needed in order to test this premise however.

It may seem difficult for one to reconcile that acute provision of CaHMB, in the absence of exogenous nutrition (i.e. EAA's) and following an overnight fast, is still able to elicit a robust, perhaps near maximal stimulation of MPS, i.e. raising the question as to where the additional AA's substrates required for supporting this MPS response are coming from. It would appear that the AA's to support this response are derived from endogenous intracellular/plasma pools and/or protein breakdown (which will increase in fasted periods). This is not as unusual a phenomenon as one may expect. Indeed, MPS, over equivalent acute time period of a few hours, can be stimulated to near maximal levels by boluses of single EAAs [5], [37] (and consequently depleting intramuscular BCAA concentrations), insulin (where MPB will be inhibited too thereby reducing endogenous AA pool size [38]) and acute resistance exercise [39]; all in the absence of exogenous nutritional intake. This clearly highlights that there are sufficient stores of AA's to support *acute* short term increases in MPS in an experimental setting; this may of course differ in a perpetually undernourished or fasted state. Moreover, it should be noted however that the premise of HMB as a nutritional supplement is not meant as a replacement for standard nutrition, but as an adjuvant therapy to support and maximize maintenance of muscle mass alongside optimal nutritional intake.

There is further evidence that the molecular regulation of HMB's effects on MPS are occurring via activation of mTORc1, with increases in phosphorylation seen in downstream substrates, p70S6K1 and RPS6 (Fig. 3). This agrees with our previous data with HMB in its FA form [8], which saw similar molecular responses, as well as that provided by preclinical work [13]. Unexpectedly however, there was a decrease in the phosphorylation of Akt at both Ser308 and Thr473, which may seem counterintuitive based on the role of Akt in the upstream activation of mTOR [40]. However, there is evidence that hyper-active mTOR negatively feeds back to reduce Akt phosphorylation [41]; therefore, the potent increase in p70S6K and RPS6 phosphorylation may have in turn caused downregulation of Akt through this negative feedback loop. Moreover, we hypothesise (as with leucine [42]), HMB's effects on mTORc1 are downstream of Akt.

The nutritional regulation of MPB is normally driven via an insulin dependent effect, whereby secretion of insulin via carbohydrate and/or protein intake provide a nitrogen sparing effect thereby promoting positive net balance [6]. However, HMB has previously been shown to have no effect on insulin [8]; as such, suppression of MPB could be considered insulin independent. Indeed, HMB has been shown to have anti-catabolic properties in pre-clinical models, through action to suppress ubiquitin – proteasomal [43], [44] and autophagy [45] pathways following pharmacological enhancement of proteolysis. Yet despite an effect on MPB here, there were no obvious effects on the atrogenes MuRF1 and MAFbx (see Fig. 3E and F), therefore, how and through which proteolytic pathway HMB is regulating reductions in MPB *in vivo* is as yet not clear. However, we have previously reported that the acute suppression of MPB during insulin clamped conditions are not associated with altered abundance of a number of proteolytic markers such as MuRF1 and Mafbx [6], [46], therefore our observations here with proteolytic targets may not be so unexpected. Moreover, it is possible that with only a single timepoint, peak signalling events in response to HMB may not have been possible to capture – an inherent limitation of clinical studies – in order to understand the key signalling mechanisms involved in the modulation of MPS and MPB by HMB, a timecourse study is needed. Further mechanistic research is needed to resolve the mechanisms underlying the acute anti-proteolytic effects of HMB in skeletal muscle.

To conclude, a large single oral dose (∼3 g) of Ca-HMB robustly (near maximally) stimulates skeletal muscle anabolism, in the absence of additional nutrient intake; the anabolic effects of Ca-HMB are equivalent to FA-HMB, despite purported differences in bioavailability (Fig. 4). This data suggests the threshold for stimulation of MPS by HMB is likely to be lower than thought, further highlighting the potent anabolic properties of HMB and supporting its use as a pharmaconutrient in the modulation muscle mass in health and disease. Future research should determine whether this acute anabolic response can be re-capitulated over more chronic periods (using novel techniques such as D_2_O stable isotope tracer techniques) alongside standard nutrition, and in groups of individuals where such benefits may be most useful (i.e. ageing and non-communicable disease states). In addition, the efficacy of HMB would need to be tested using the gold standard placebo controlled designed, something that could be considered a limitation in the present study. Moreover, investigation as to whether there is a distinct threshold for the efficacy of HMB (in either form) through a dose response approach, will also help clarify whether there is a relationship between plasma bioavailability and the positive anabolic effects of HMB.

## Statement of authorship

PJA, JAR, KS, NJS, JW & JL conceived and designed the study. TH & ML performed all data collection. DJW, BEP, JC, ML, MSB, AP & SA performed the sample processing, data analyses and construction of figures. All authors contributed to the preparation and drafting of the final manuscript.

## Conflict of interest

JAR is an employee of Metabolic Technologies Inc. All other authors state no conflict of interest.

## Funding sources

This work was supported by an unconditional grant from Metabolic Technologies Inc; the Medical Research Council [grant number MR/K00414X/1]; and Arthritis Research UK [grant number 19891]. DJ Wilkinson was a post-doctoral research fellow funded through the MRC-ARUK Centre for Musculoskeletal Ageing Research awarded to the Universities of Nottingham and Birmingham. Metabolic Technologies Inc. supplied the Ca-HMB on a collaborative basis and undertook the HMB plasma analyses, but were blinded to the sample identities. A Philp is supported by BBSRC New Investigator Award [grant number BB/L023547/1].
